# Non-Invasive Assessment of the Interrelationships of Diet, Pregnancy Rate, Group Composition, and Physiological and Nutritional Stress of Barren-Ground Caribou in Late Winter

**DOI:** 10.1371/journal.pone.0127586

**Published:** 2015-06-10

**Authors:** Kyle Joly, Samuel K. Wasser, Rebecca Booth

**Affiliations:** 1 Gates of the Arctic National Park & Preserve, National Park Service, Fairbanks, Alaska, United States of America; 2 Arctic Inventory and Monitoring Network, National Park Service, Fairbanks, Alaska, United States of America; 3 University of Washington, Center for Conservation Biology, Seattle, Washington, United States of America; Institut Pluridisciplinaire Hubert Curien, FRANCE

## Abstract

The winter diet of barren-ground caribou may affect adult survival, timing of parturition, neonatal survival, and postpartum mass. We used microhistological analyses and hormone levels in feces to determine sex-specific late-winter diets, pregnancy rates, group composition, and endocrine-based measures of physiological and nutritional stress. Lichens, which are highly digestible but contain little protein, dominated the diet (> 68%) but were less prevalent in the diets of pregnant females as compared to non-pregnant females and males. The amount of lichens in the diets of pregnant females decreased at higher latitudes and as winter progressed. Pregnancy rates (82.1%, 95% CI = 76.0 – 88.1%) of adult cows were within the expected range for a declining herd, while pregnancy status was not associated with lichen abundance in the diet. Most groups (80%) were of mixed sex. Male: female ratios (62:100) were not skewed enough to affect the decline. Levels of hormones indicating nutritional stress were detected in areas of low habitat quality and at higher latitudes. Levels of hormones indicated that physiological stress was greatest for pregnant cows, which faced the increasing demands of gestation in late winter. These fecal-based measures of diet and stress provided contextual information for the potential mechanisms of the ongoing decline. Non-invasive techniques, such as monitoring diets, pregnancy rates, sex ratios and stress levels from fecal samples, will become increasingly important as monitoring tools as the industrial footprint continues to expand in the Arctic.

## Introduction

The cumulative effects of industrial development are impacting caribou (*Rangifer tarandus*) populations, especially in the southern portions of their range. These impacts have been primarily documented in boreal caribou populations, while many barren-ground caribou herds in the arctic may be declining as well [1]. Industrial development is increasing in the Arctic and has the potential to have similarly negative consequences for barren-ground caribou as has been documented for boreal caribou [2–4]. Aside from habitat degradation and conversion, displacement of caribou from industrial development has received the most attention (*e*.*g*. [5]). However, industrial development may have numerous other indirect impacts on caribou populations, such as increasing nutritional stress and altering predator-prey dynamics [6,7].

Caribou, ranging from temperate rainforests to polar desert, are capable to adapting to a wide array of conditions that climate change may induce. Some potential changes, such as increased vegetative productivity in the Arctic, may actually prove beneficial for caribou. However, other changes, such as increased occurrence of icing events [8] and conversion of tundra to shrub habitats [9] may prove detrimental [10,11]. A warmer Arctic may lead to drier conditions and more wild fires which, in combination with competition from expanding shrub habitats, could lead to declines in lichen abundance [12].

Terricolous lichens are an important component of the winter diets of barren-ground caribou [13– 17]. Lichens are rich in digestible energy but low in protein [18]. The availability of lichens throughout the winter reduces the dependence of female caribou on body reserves that are important to survival [19] and reproduction [20]. Snow depth and hardness affects the availability of lichens for caribou [21].

Winter nutrition is linked with adult survival, timing of parturition, neonatal survival, and postpartum mass [22–27]. Low neonatal survival, resulting from poor winter nutrition during late gestation, could incorrectly be attributed to predation [27]. Predation on neonates could similarly obfuscate the effects of nutritional stress if high pregnancy rates exist [27]. Depressed postpartum mass could affect milk production and place cows at a disadvantage to reach an adequate nutritional plane to conceive in the fall [27,28]. Caribou may be able to compensate over the summer given favorable summer range conditions, however, Dale *et al*. [29] found that compensatory growth in smaller individuals occurred during winter months, not summer, for young caribou.

The Western Arctic Herd, numbering 490,000 caribou in 2003, was the largest herd in Alaska and one of the largest in the world [30]. Rural residents from about 40 villages in northwest Alaska rely upon these caribou as a critical subsistence resource. Since 2003, the herd has rapidly declined [30] to 235,000 caribou in 2013 [31]. Coupled with the undetermined mechanism for the decline, the uncertain effects of a rapidly changing climate [10,32] and proposed industrial development in the region [4,33] are amplifying concerns about the population as well as the availability of caribou for subsistence-based communities in the region. Therefore, to establish a baseline for monitoring, we used a non-invasive fecal sampling approach and endocrine-based measures of stress to determine and evaluate the interrelationships of winter diets, sex, pregnancy status, group composition, and physiological and nutritional stress in overwintering caribou. While an analogous effort was completed for boreal caribou in Alberta [7], harsh environmental conditions, massive caribou ranges, and difficult logistics have prevented a similar effort for barren-ground caribou in the Arctic.

## Material and Methods

### Study Area

**Fig 1:**
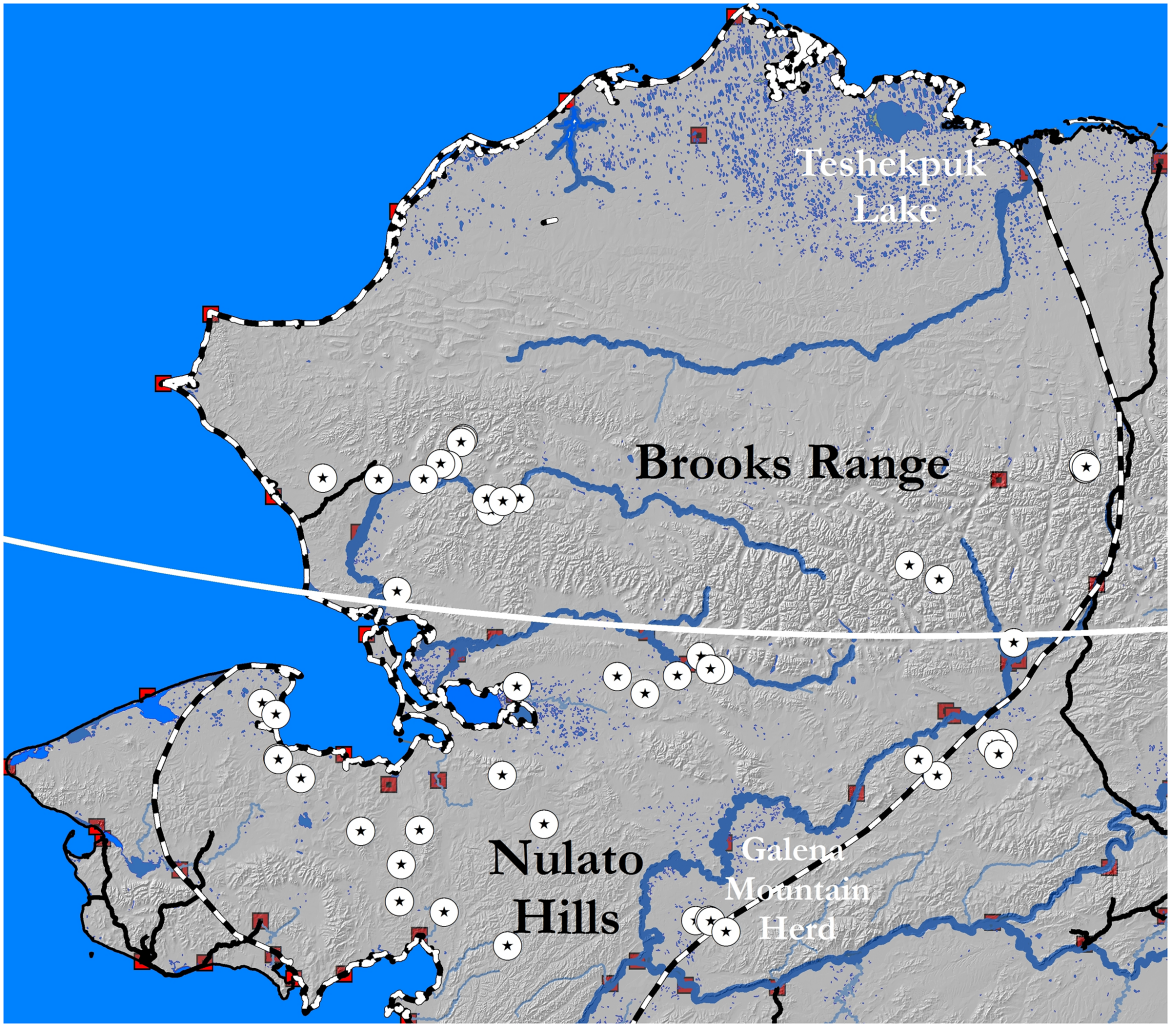
Study area of northwest Alaska, 2012–2013. White circles with black stars inside represent fecal pellet sample site locations. The black and white dashed line represents the extent the Western Arctic Herd range. In 2012, caribou moved outside this boundary to the southeast. White line (67.1° N) generally separates sites south of the Brooks Range from those within it, dark squares are villages, and black lines are roads.

The study area included the entire winter range of the Western Arctic Herd, covering over 360,000 km^2^ (Fig 1). The vast region ranges from coastal to continental climates of the arctic and subarctic with expanses of tundra, taiga, wetlands and mountains. For more details about the study area, see Joly *et al*. [34,35]. Caribou from the Teshekpuk Lake Herd can utilize the same winter ranges as the Western Arctic Herd [36] and Central Arctic Herd caribou winter range can overlap the northeast portion of the Western Arctic Herd’s range [30]. Thus, results from most of the fecal analyses were not assigned to any particular herd but rather simply designated ‘arctic caribou’. Samples from 4 sites east of the Nulato Hills (Fig 1) were from the small (~ 400 total individuals) resident Galena Mountain Herd (GMH) [30]. In spring 2012, some Western Arctic Herd caribou were found southeast their typical winter range (Fig 1), an area that they had not utilized in large numbers for a decade, perhaps due to deep snow conditions that occurred during the winter of 2011–2012.

### Fecal Pellet Collection

We collected 221 fecal samples from 38 locations between March 22 and April 27, 2012 and 130 samples from 18 locations between April 15 and April 17, 2013 across northwest and north-central Alaska (Fig 1). Locations were primarily accessed by small ski-equipped planes (*e*.*g*., Piper PA-18 Super Cub), though some were by dog team or snowmachine, immediately after spotting caribou to collect fresh samples. Ten to 15 pellets were collected from distinct fecal groups, placed in a plastic bag, and kept frozen until processing. Mean number of samples (i.e., fecal groups) collected per location was 6 (range 1–16). Locations were not revisited and were spatially separated so that caribou were not resampled. The smaller pellets typical of calves were not collected. While impossible to rule out, the collection of multiple samples from a single individual was unlikely due to the spatial and temporal distribution of sample locations and number of caribou at each sample location.

### Microhistology

All 221 samples collected in 2012 were submitted for microhistological diet analysis, but none from 2013 due to budgetary restraints. All samples were analyzed by the same laboratory to minimize sources of error [14]. Relative density of plant fragments was based on 100 views per sample (Level B) and corrected for apparent digestibility according to Boertje [37] and Gustine *et al*. [38].

### DNA and Hormone Analyses

Fecal samples were swabbed with buccal swabs to obtain DNA samples from the mucosal cells on their surfaces. The DNA was extracted from the swab using a silica-based purification system and used to determine sex, following the techniques outline in Wasser *et al*. [7,39] and Ball *et al*. [40]. DNA extracts underwent polymerase chain reaction (PCR) for amplification, PCR products were separated using an ABI 3730 Genetic Analyzer, and visualized using GeneMarker software. Homozygotes (i.e., female, X/X) were confirmed if the X chromosome alone was seen at least 3 times, whereas heterozygotes (i.e., males, X/Y) were confirmed if the Y chromosome was seen at least twice. A portion of each sample was homogenized and freeze-dried and then ground to a fine powder. A pulse-vortex double extraction with 70% ethanol was performed and then radioimmunoassays (RIA) were conducted at previously validated dilutions for fecal metabolites of progesterone, glucocorticoid (GC; cortisol), and the thyroid hormone triiodothryronine (T3) [7]. We categorized caribou with > 2000 ng/g progesterone as pregnant [41]. Immature caribou (< 4 years old) are known to have lower pregnancy rates than mature (> 3 years old) cows [27,42–45]. Therefore, based on these publications, we estimated that proportion of breeding females was 85% for this steadily declining arctic caribou and used this estimate to recalculate the pregnancy rate.

### Geospatial and Statistical Analyses

We determined distance to nearest village for every sample location using ArcGIS (ESRI, Redlands, CA). We used linear regression techniques to identify relationships between continuous variables: 1) proportion of lichen in the diet and latitude and Julian day, and 2) hormone levels and distance to villages. For comparisons among pregnant females, non-pregnant females and males, and hormone levels, we utilized analysis of variance (ANOVA). We employed second-order polynomial regression analysis to assess the relationships between latitude and cortisol and T3 levels [46].

### Ethics Statement

Permission to collect fecal samples was obtained from Noatak National Preserve, Gates of the Arctic National Park, Bering Land Bridge National Preserve, Selawik National Wildlife Refuge, Kanuti National Wildlife Refuge, and Koyukuk National Wildlife Refuge. Sampling procedures were approved as part of getting permission to collect samples. To our knowledge, permission to collect fecal samples was not required at locations outside of these areas. Field studies did not include endangered or protected species. An Institutional Animal Care and Use Committee (IACUC) review was not required for our research as no animals were handled.

## Results

We were able to definitively identify the sex of the caribou from 332 of the 351 samples. Group composition ranged from all males to all females, typical of sexual segregation during winter. However, of the 45 locations that had >1 sample, 80% of the groups contained both bulls and cows. For the single-sex groups (n = 11), diet did not significantly vary between female-only and male-only groups. The sex ratio for arctic caribou (n = 301) was 62 males: 100 female (95% CI 49–78 males: 100 females).

Overall, 134 of 195 cows were pregnant (68.7%; 95% CI 62.2–75.3%). However, only 50.0% (16.8–83.2%) the GMH cows (n = 12) were pregnant as, compared to 70.0% (63.2–76.7%) for the arctic caribou (n = 183). Pregnancy rate, by site, averaged 69.8% (SE = 5.1. Correcting (see Methods) for lower reproductive rate of young (1.8–3.8 years of age), pregnancy rate of mature arctic caribou cows was 82.1% (76.0–88.1%).

**Fig 2:**
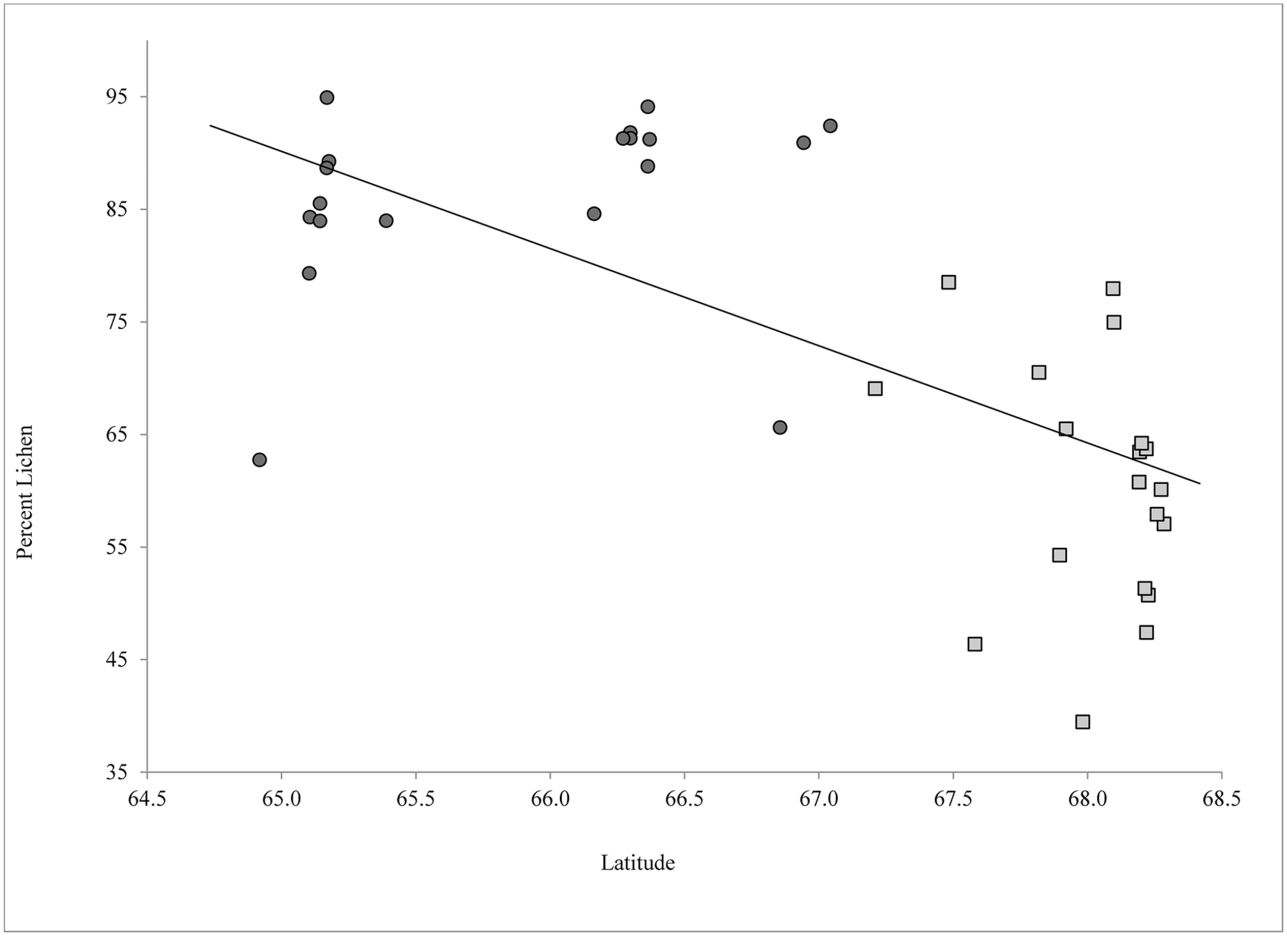
The amount of lichen (%) in the diets of caribou in northwest Alaska relative to latitude (° N). Dark circles are south of the Brooks Range (67.1° N) and lighter squares are within the range (north of 67.1° N).

Caribou diets varied between sexes and pregnancy status (Table 1A). Lichens were the predominant forage for all caribou, yet pregnant caribou had less lichen (70.1%; 95% CI 66.5–73.6%) in the diet than non-pregnant caribou (75.8%; 95% CI 70.1–81.6%) and males (75.9%; 95% CI 72.8–79.0%; F_2, 206_ = 3.49, P = 0.03; Table 1). Diets of GMH caribou had more lichen than the arctic caribou (Table 1B and 1C). The amount of lichen in the diet of caribou at a given location declined with increasing latitude (Fig 2; R^2^ = 0.482, F = 33.46, df = 36, P < 0.01). Pregnancy was not associated with latitude or with the amount of lichen found in the diet, however, for pregnant cows, the amount of lichen in the diet was negatively related to Julian day (*i*.*e*., less lichen in the diet as the year progressed; R^2^ = 0.18, F = 17.01, df = 79, P < 0.01). This relationship held for non-pregnant cows (R^2^ = 0.17, F = 6.75, df = 33, P = 0.01) and bulls (R^2^ = 0.09, F = 9.17, P < 0.01).

**Table 1:** Diet composition (mean ± SE %) of caribou in northwest Alaska derived from microhistology of feces collected in 2012 that were corrected for digestibility (*sensu* Gustine *et al*., 2011).

A. All Samples							
Class	N	Shrub	Forbs	Gram	Lichen	Misc	Moss
Pregnant Cows	81	7.7 (0.6)	2.9 (0.5)	6.1 (0.4)	70.1 (1.7)	0.0 (0.0)	13.2 (1.0)
Non-Pregnant Cows	34	6.0 (0.9)	2.3 (0.7)	4.5 (0.7)	75.8 (2.7)	0.1 (0.0)	11.4 (1.5)
Bulls	92	7.0 (0.6)	1.5 (0.5)	5.0 (0.4)	75.9 (1.6)	0.2 (0.0)	10.5 (0.9)
**B. Arctic caribou**							
Class	N	Shrub	Forbs	Gram	Lichen	Misc	Moss
Pregnant Cows	75	8.2 (0.6)	3.1 (0.7)	6.3 (0.4)	68.4 (1.8)	0.0 (0.0)	14.0 (1.0)
Non-Pregnant Cows	28	6.8 (1.0)	2.6 (0.7)	5.2 (0.5)	72.5 (2.9)	0.1 (0.0)	13.0 (1.7)
Bulls	74	7.6 (0.7)	1.7 (0.3)	5.9 (0.5)	72.8 (1.7)	0.1 (0.0)	11.9 (1.0)
**C. Galena Mountain Herd caribou**							
Class	N	Shrub	Forbs	Gram	Lichen	Misc	Moss
Pregnant Cows	6	2.1 (0.9)	0.8 (0.5)	3.3 (1.7)	90.5 (2.2)	0.1 (0.1)	3.1 (1.5)
Non-Pregnant Cows	6	2.2 (0.7)	1.1 (1.0)	1.0 (0.5)	91.5 (4.6)	0.3 (0.2)	3.9 (2.5)
Bulls	18	4.2 (0.5)	0.4 (0.1)	1.6 (0.4)	88.8 (1.4)	0.4 (0.1)	4.7 (1.0)

‘Gram’ is graminoids, which includes grasses and sedges. ‘Forbs’ includes *Equisetum* spp. ‘Misc’ is miscellaneous which is comprised mostly of items difficult to digest such as seeds and spruce needles.

**Fig 3:**
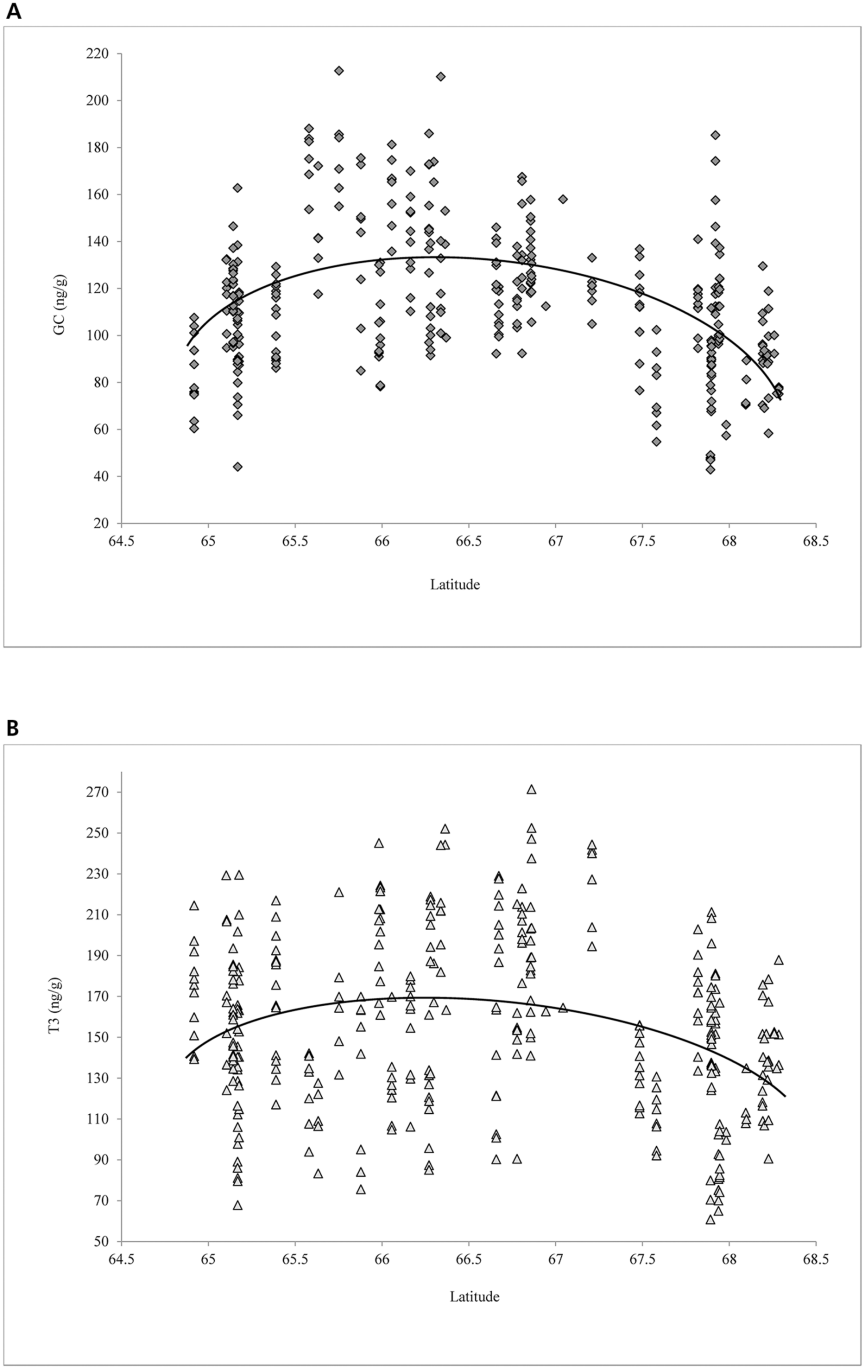
Levels (ng/g) of hormones derived from analyses of caribou fecal material, northwest Alaska, March and April, 2012–2013. (A) Glucocorticoid (GC) and (B) Triiodothryronine (T3).

Individual progesterone levels ranged from 67.5–18603.0 ng/g, while cortisol and T3 levels ranged from 42.8–212.6 ng/g and 0.0–271.4 ng/g, respectively. Both cortisol and T3 levels for sites were associated with latitude (R^2^ = 0.46, F = 23.01, df = 53, P < 0.01; R^2^ = 0.22, F = 7.59, df = 53, P < 0.01, respectively), with highest levels occurring at mid-latitude sites (Fig 3). Both hormones decreased with increasing distance from the nearest town (R^2^ = 0.21, F = 14.78, df = 54, P < 0.01; R^2^ = 0.10, F = 5.79, df = 54, P = 0.02, respectively). Cortisol levels differed (F_2, 325_ = 3.28, P = 0.04) among pregnant cows, non-pregnant cows, and bulls with the highest levels for pregnant cows and lowest for bulls (Table 2). For the 11 single-sex groups, cortisol was higher (F_2, 325_ = 7.93, P = 0.02) in cow-only groups ($\bar{x}=134.8$, SE = 10.0) than in bull-only groups ($\bar{x}=93.1$, SE = 11.0). Cortisol levels were higher (F_1, 347_ = 23.75, P < 0.01) for caribou found at the southeastern fringe ($\bar{x}=143.3$, SE = 6.2) of the herd’s range in 2012 than elsewhere ($\bar{x}=112.2$, SE = 1.6).

**Table 2:** Hormone levels (mean ± SE ng/g) of caribou in northwest Alaska in 2012 and 2013.

A. All Samples				
Class	N	Progesterone	Glucocorticoid (GC)	Triiodothryronine (T3)
Pregnant Cows	134	8531.7 (354.2)	118.5 (2.8)	156.2 (3.4)
Non-Pregnant Cows	61	674.7 (164.1)	112.1 (4.3)	151.7 (6.2)
Bulls	131		109.1 (2.3)	153.2 (4.0)
**B. Arctic caribou**				
Class	N	Progesterone	Glucocorticoid (GC)	Triiodothryronine (T3)
Pregnant Cows	128	8386.0 (362.1)	119.7 (2.9)	158.7 (3.3)
Non-Pregnant Cows	55	731.2 (180.5)	114.5 (4.4)	151.4 (6.4)
Bulls	114		109.4 (2.6)	153.2 (4.5)
**C. Galena Mountain Herd caribou**				
Class	N	Progesterone	Glucocorticoid (GC)	Triiodothryronine (T3)
Pregnant Cows	6	11641.0 (1208.7)	93.3 (5.4)	102.8 (10.7)
Non-Pregnant Cows	6	157.0 (21.0)	90.0 (14.8)	154.7 (23.5)
Bulls	17		107.1 (4.8)	153.3 (9.2)

## Discussion

We conducted, to our knowledge, one of the most extensive microhistological analyses of the winter diets of barren-ground caribou to date—although it was limited to late winter (March/April) in a single year. In conjunction with DNA and hormone level analyses, we were able to differentiate diets of pregnant and non-pregnant cows from bulls. In concurrence with other studies [15–17,37,47,48], we found lichens constituted the majority of the diet for vast majority of sampled caribou, with some individuals having more than 90% in their late-winter diet. There were broad similarities in the diets of pregnant cows, non-pregnant cows and bulls; however pregnant arctic caribou cows had less lichens and more shrubs, forbs and graminoids in their diet (Table 1). Each of these vascular forage classes contain more N than lichens [47]. While the differences are small, pregnant cows may have adjusted their diet to compensate for the additional protein demand of gestation [49,50]. Additionally, as winter progressed, the amount of lichen declined in the diets of all caribou which may have reflected a decline in the availability of lichen, an increase in the availability of other forages as snow melted, and/or a shift to vascular forages to acquire protein [16].

As expected, the amount of lichen in the diet was not associated with the likelihood of being pregnant. Pregnancy is largely a factor of autumn weight/body condition [26,27,51]. Pregnancy rate (95% confidence interval = 76–88%) was slightly lower than what would be expected for a healthy, increasing herd (~90%) [44,52–54]. Given the Western Arctic Herd’s high population size and decade-long decline, the lower pregnancy rate is not unexpected [27,55]. The lower pregnancy rate may suggest that summer-autumn range conditions may be suboptimal, as found with woodland caribou [56]. High caribou density could exacerbate summer range conditions already stressed by low productivity that was facilitated by climatic conditions associated with a phase change in the Pacific Decadal Oscillation [57]. However, the difference in pregnancy rate is most likely not large enough to account for the herd’s decline.

Lichens are highly digestible and high in carbohydrates, but low in N [18]. This makes lichens a good source of energy for caribou, perhaps facilitating overwinter survival and/or lipogenesis [58]. Lichen abundance is typically lower in the northern most portions of Alaska [13,59]. The amount of lichen in the diet declined significantly the further north caribou overwintered. This trend (Fig 2) was even more distinctive when the mean dietary lichen for caribou residing south of the Brooks Range (~ 67.1° N; 87.3% ± 2.1) was compared with those caribou within and north of the range (60.8% ± 2.0). One departure from this trend was at the southernmost location (64.9° N) in the Nulato Hills region (Figs 1 and 2), which has received heavy winter use for the past decade [34,35], where mean dietary lichen was 62.7% ± 2.3. Although the herd’s core winter range has been grazed intensively, caribou are still capable of finding lichens in comparable amounts to other herds [17]. Heavy use has, however, led to an expansion of the Western Arctic Herd’s winter range thereby possibly forcing animals to habitats of varied quality [35], increasing energetic costs of acquiring forage [60], and/or exposure to higher levels of predation [61]. Despite lower snow depths and predator abundance [59], Western Arctic Herd caribou that overwinter north of the traditional wintering grounds tend to survive winter at lower rates than those that migrate (Joly, unpublished data).

Spatial or dietary segregation between males and females was minimal. We found that 80% of the groups were of mixed sex, with the remainder being all bulls or all cows. Sexual segregation is common but highly variable in barren-ground caribou and can occur throughout the year [62]. Winter is a time of energy and protein conservation for caribou, which may lead to physiological convergence between large and small individuals and among various sex and age classes (*i*.*e*., relatively larger individuals losing more mass than smaller ones) [28,29,58]. Body mass differences between males and females suggest males can subsist on lower quality diets [63]; however, the diets of all bull and all cow groups were not different. Additionally, the preponderance of mixed sex groups throughout the winter range could make spring composition counts more difficult. Selection of higher and steeper terrain by bulls [59], which would be harder to access by ski plane, dog team or snowmachine, could mask sexual segregation, as determined from fecal analyses, at this time of year. However, if this were the case, our bull: cow would be even higher.

We determined a sex ratio of 62 males: 100 females for arctic caribou, which is higher than the estimated declining ratio of < 50 reported for the WAH for the past decade [30]. Barren-ground caribou are highly polygynous. Therefore, we suspect, even at the lower reported levels, that bull: cow ratios are not impacting herd productivity at this time.

T3 (triiodothryronine), a hormone negatively associated with nutritional stress [64], was high (*i*.*e*., low stress) at mid-latitudes (Fig 3). Dietary lichen abundance was not associated with T3 levels. While speculative, the combination of higher T3 (*i*.*e*., lower nutritional stress) and cortisol levels (*i*.*e*., higher physiological stress) [65,66] near villages is suggestive of caribou taking higher risk of and/or enduring higher stress from human predation/disturbance for access to better forage near villages. However, non-human predators are also typically at lower densities near villages (Joly, personal observation). Low T3 levels (*i*.*e*., higher nutritional stress) were found in samples nearest (within 16 km) the Red Dog Mine road—an area where vegetation has been impacted by road dust [67].

Not surprisingly, cortisol was higher in pregnant cows and at mid-latitudes for all caribou. Although the costs of gestation are minimal in early pregnancy, they increase substantially during the third trimester when samples were obtained [50,68]. Greater density of overwintering caribou is the most parsimonious rationale for the higher physiological stress in this region, though greater non-human predator abundance is a possible factor leading as well. Unfortunately, data on predator abundance in this region is scarce. The combination of higher cortisol at mid-latitude and near villages supports the hypothesis that human predation pressure can increase physiological stress in caribou. We also found high cortisol levels in caribou that overwintered in areas of deep snow, outside their most commonly used wintering grounds. The cortisol levels we detected were higher than those reported for captive caribou and reindeer [66,69], similar to caribou in Alberta [7], but less than those subjected to a hormone challenge [66]. Our hormone analyses are the first of their kind in the region and should be viewed cautiously as a pre-development baseline reference.

## Conclusion

Though abundant in the diet of overwintering barren-ground caribou, the amount of lichen was not related to pregnancy rate, as expected, or level of nutritional stress. We found nutritional stress, as indexed by low T3 levels, was greatest at high latitude where habitat quality and lichen abundance was low. Given evidence that Western Arctic Herd caribou that overwinter north of their traditional wintering grounds have lower survivorship (Joly, unpublished data), we put forth the hypothesis that relative importance of lichens in the diet of overwintering caribou is related more to adult survivorship than pregnancy and parturition in our study area. This could manifest itself through better body condition to evade predators. Aside from evidence that caribou wintering north of the herd’s core winter range fare poorer, we did not detect robust, widespread indications of winter time malnutrition. Low winter food availability during late gestation could lead to lower neonatal survival [27] and thus recruitment, which has been detected for the Western Arctic Herd [30]. Suboptimal summer range conditions may lead to depressed milk output, less robust calves, less cows being pregnant and lower survival rates. We found slightly lower than expected pregnancy rates and concurrent with high levels of adult cow mortality during summer (June, July, August; Joly, unpublished data). Additionally, poor recruitment of calves has been documented [30]. Reduced reproductive output and recruitment in concert with lower adult survival rates could be facilitating the herd’s decline and should be investigated further before additional development occurs.
